# Connexin 43 Differentially Regulates Epileptiform Activity in Models of Convulsive and Non-convulsive Epilepsies

**DOI:** 10.3389/fncel.2019.00173

**Published:** 2019-05-10

**Authors:** Renáta Vincze, Márton Péter, Zsolt Szabó, Julianna Kardos, László Héja, Zsolt Kovács

**Affiliations:** ^1^Functional Pharmacology Research Group, Institute of Organic Chemistry, Research Centre for Natural Sciences, Hungarian Academy of Sciences, Budapest, Hungary; ^2^Department of Biology, Eötvös Loránd University, Savaria University Centre, Szombathely, Hungary

**Keywords:** epilepsy, temporal lobe epilepsy, absence epilepsy, astrocytes, gap junctions, neuro-glia interaction, astrocyte synchronization, astrocyte network

## Abstract

The influence of astrocytic cell networks on neuronal network activity is an emerging issue in epilepsy. Among the various mechanisms by which astrocytes modulate neuronal function, synchronization of astrocytes via gap junction channels is widely considered to be a crucial mechanism in epileptic conditions, contributing to the synchronization of the neuronal cell networks, possibly inducing recurrent epileptiform activity. Here, we explored whether modulation of astrocytic gap junctions could alter epileptic seizures in different types of epilepsy. Opening of gap junctions by trimethylamine intensifies seizure-like events (SLEs) in the low-[Mg^2+^] *in vitro* model of temporal lobe epilepsy, while alleviates seizures in the *in vivo* WAG/Rij rat model of absence epilepsy. In contrast, application of the gap junction blocker carbenoxolone prevents the appearance of SLEs in the low-[Mg^2+^] epilepsy model, but aggravates seizures in non-convulsive absence epilepsy, *in vivo*. Pharmacological dissection of neuronal vs. astrocytic connexins shows that astrocytic Cx43 contribute to seizure formation to a significantly higher extent than neuronal Cx36. We conclude that astrocytic gap junctions are key players in the formation of epileptiform activity and we provide a scheme for the different mode of action in the convulsive and non-convulsive epilepsy types.

## Introduction

Epilepsy is one of the most common neurological disorder affecting 1–2% of the population worldwide. It is considered to be an enhanced neuronal activity characterized by spontaneous recurrent seizures, which can be prevented by appropriate medication only in 70% of patients, but one-third remains pharmacoresistant (Eadie, 2012). The epilepsy-associated genes identified so far encode mostly ion channels and ligand gated glutamate (Glu) and γ-amino-butyric acid (GABA) neurotransmitter receptors, which are the main targets of anti-epileptic drugs developed up to date (Verkhratsky and Steinhäuser, 2000; Steinlein, 2004; Mulley et al., 2005; Berkovic et al., 2006; Weber and Lerche, 2008). Due to the high incidence of pharmacoresistance and serious side effects, however, disclosing potential new target mechanisms is crucially important in order to serve more effective anti-epileptic drug development campaigns.

Glial cells have so far been ignored in epileptogenesis, but many recently revealed mechanisms support the notion that besides neurons, glial cells (predominantly astrocytes) are also significant contributors of epileptic activity (Verkhratsky et al., 2013; Héja, 2014; Kékesi et al., 2015). In addition to classical functions, like K^+^ buffering through Na^+^/K^+^ pumps, Na^+^/K^+^/Cl^–^ cotransporters and Kir channels (Kofuji and Newman, 2004; Wallraff et al., 2006; Seifert et al., 2009; Bedner and Steinhäuser, 2013; Mukai et al., 2018), water regulation by aquaporin-4 channel (Binder et al., 2012; Hubbard et al., 2015) as well as glutamate uptake *via* two astrocytic excitatory amino acid transporter subtypes, EAAT1 and EAAT2 (Watanabe et al., 1999; Héja, 2014; Eid et al., 2018), astrocytes can directly modulate synaptic activity. They can dynamically interact with neurons by releasing various neurotransmitters (Glu, GABA, D-serine, ATP) in response to intracellular Ca^2+^ concentration changes triggered by ionotropic and metabotropic receptor activation (Seifert et al., 2009; Héja, 2014; Steinhäuser et al., 2016; Robin et al., 2018). Moreover, astrocytes can even turn glutamatergic excitation into tonic inhibition by the Glu/GABA exchange mechanism (Héja et al., 2009, 2012).

Furthermore, astrocytes are able to establish direct physical contacts through gap junction channels, composed of connexin proteins (Héja, 2014; Beyer and Berthoud, 2018; Beyer et al., 2018). These transmembrane channels can synchronize electric and metabolic activities of astrocytes by the direct exchange of ions and small molecules, like Ca^2+^, IP_3_, cyclic AMP, and oligonucleotides (Beyer et al., 2018). In addition to forming intercellular coupling, connexin hemichannels can also release ATP that consequently activates purinergic receptors on neighboring astrocytes, thereby establishing indirect synchronization routes as well (Guthrie et al., 1999; Bennett et al., 2003). Utilizing these pathways, gap junction proteins participate in the propagation of Ca^2+^ waves (Cotrina et al., 1998; Rouach et al., 2008) that are associated with the long-range coupling of the astrocytic syncytium and the synchronization between astrocytes and neuronal populations in epilepsy (Héja, 2014; Kékesi et al., 2015). We have previously confirmed that this long-range synchronized Ca^2+^ signaling might have proepileptic effects, since it contributes to the appearance of seizure-like events (SLEs) (Kékesi et al., 2015). Increased glucose uptake associated with the inhibition of gap junction coupling also supports this assumption (Tabernero et al., 2006), because intercellular trafficking of glucose and other energetic metabolites maintains synaptic transmission under pathophysiological conditions such as epilepsy (Rouach et al., 2008; Seifert et al., 2009; Bedner and Steinhäuser, 2013). These findings confirmed the fact that besides their abovementioned antiepileptic actions, such as Glu uptake and K^+^ redistribution, astrocytes may also significantly contribute to the formation of seizures. Moreover, Glu, released by hemichannels can activate extrasynaptic Glu receptors, eventually increasing overall excitation level. To ensure more effective anti-epileptic drug development, it is important to examine the role of astrocytes in different epilepsy models.

Excessive neuronal synchronization and excitability have a role in the generation of mechanistically different epilepsy types, like the temporal lobe epilepsy (TLE) and absence epilepsy (Coenen and Van Luijtelaar, 2003; Avoli, 2014). As astrocytic synchronization may be one of the main seizure initiating factors, and may have a role in the neuronal synchronization, we hypothesized that alterations in astrocytic synchronization by modulation of gap junction channels may affect epileptiform activity in both TLE and absence epilepsy.

Accordingly, our aim was to investigate the role of gap junctions and the astrocytic syncytium in two different epilepsy models, the *in vitro* low-[Mg^2+^] model (*in vitro* model of TLE) and *in vivo* in the animal model of human absence epilepsy Wistar Albino Glaxo/Rijswijk (WAG/Rij) rats (Coenen and Van Luijtelaar, 2003). In contrast to the convulsive TLE, WAG/Rij rats show non-convulsive type of epilepsy, developing secondary generalized seizures, the bilaterally synchronous spike-wave discharges (SWDs) (Coenen and Van Luijtelaar, 2003; Kohmann et al., 2016). Convulsive and anticonvulsant effects of gap junction opener and blocker trimethylamine hydrochloride (TMA) and carbenoxolone hemisuccinate (CBX), respectively, have already been demonstrated in TLE models *in vivo* (Gajda et al., 2003). Therefore, we asked if gap junction communication shaping TLE could also be pharmacologically isolated by comparing effects of TMA and CBX in the low-Mg^2+^ model of TLE *in vitro*. Inhibitory CBX effects have been reported in human (Gigout et al., 2006) and animal absence epilepsies (Gareri et al., 2005) as well. However, we have no data on TMA effects in the absence epilepsy; thus, measurements on epileptic activity of WAG/Rij rats have been performed with and without TMA application. By comparison, CBX effects have also been measured.

## Materials and Methods

### Animals

Animals were kept and used in accordance with standard ethical guidelines and approved by the local Animal Care Committee, the Government Office for Pest County (Reference Nos. PEI/001/3671-4/2015 and PE/EA/3840-4/2016), the Hungarian Act of Animal Care and Experimentation (1998, XXVIII, section 243), European Communities Council Directive 24 November, 1986 (86/609/EEC) and EU Directive 2010/63/EU on the use and treatment of animals in experimental laboratories. The experiments on WAG/Rij rats were approved by the Animal Care and Experimentation Committee of the Eötvös Loránd University (Savaria University Centre) and National Scientific Ethical Committee on Animal Experimentation (Hungary) under License No. VA/ÉBNTF02/85-8/2016. All efforts were made to reduce animal suffering and the number of animals used.

Animals were housed in groups of 3–4 under standard laboratory conditions (free access to water and food; 12:12 h light–dark cycle, light was on from 08.00 AM to 08.00 PM; air-conditioned at 22 ± 2°C).

### Solutions

Artificial cerebrospinal fluid (ACSF) contained in mM: 129 NaCl; 3 KCl; 1.6 CaCl_2_; 1.8 MgSO_4_; 1.25 NaH_2_PO_4_; 21 NaHCO_3_; 10 glucose. To induce epilepsy, MgSO_4_ was eliminated and 2 mM KCl was added (low-[Mg^2+^] ACSF). In the *in vitro* experiments, CBX (200 μM), TMA (5 mM), quinine (100 μM) and La(NO_3_)_3_ were diluted in ACSF or low-[Mg^2+^] ACSF. The pH value of 7.4 was not affected by the applied TMA concentration. All solutions were continuously oxygenated (95% O_2_, 5% CO_2_). In the *in vivo* experiments on WAG/Rij rats, both CBX (100 mg/kg) and quinine (40 mg/kg) was dissolved in saline, whereas TMA (200 mM) in aseptic ACSF (Tocris, Germany). Unless otherwise stated, all drugs were purchased from Sigma-Aldrich, Budapest, Hungary.

### Slice Preparation

Transverse, 400 μm thick, hippocampal-entorhinal slices from 11 to 14 day old Wistar rats (Toxicoop, Budapest, Hungary) were prepared in modified ACSF (75 mM sucrose; 87 mM NaCl; 2.5 mM KCl; 0.5 mM CaCl_2_; 7 mM MgSO_4_; 1.25 mM NaH_2_PO_4_; 25 mM NaHCO_3_; 25 mM glucose) at 4°C. Slices were incubated in an interface-type chamber in continuously oxygenated ACSF for 1 h at 37°C followed by incubation in room temperature before performing the experiments.

### *In vitro* Electrophysiology

The electrophysiological field potential (FP) recordings were performed at 31°C. For FP recordings glass microelectrodes (1 to 4 MΩ) were filled with ACSF solution and were inserted in the CA3 *stratum pyramidale*. Signals were recorded with Multiclamp 700A amplifiers (Axon Instruments, Foster City, CA, United States), low-pass filtered at 2 kHz and digitized at 10 or 20 kHz (Digidata 1320A, Axon Instruments). Recordings were analyzed after high pass filtering at 1 or 2 Hz.

### *In vitro* Drug Application

Epileptiform activity was induced by switching the perfusing solution (ACSF) to low-[Mg^2+^] ACSF (ACSF with no added MgSO_4_ and KCl elevated to 5 mM). Drugs were applied according to two different protocols. To test the effect of the drugs on the appearance of SLEs, they were continuously present in the ACSF and low-[Mg^2+^] ACSF solutions. First, normal ACSF solution was applied for 20 min as control condition. Then normal ACSF solution with 5 mM TMA or 200 μM CBX was applied for further 20 min. Finally, low-[Mg^2+^] ACSF, containing the same concentration of drugs was applied. Another protocol was used to identify the connexin form involved in the modulation of epileptiform activity. In these experiments, drugs were applied after the detection of two fully developed SLEs. The drugs were present either during the next two SLEs or at least for 10 min (in case, the drug abolished further SLE appearances). To avoid excessive consumption of the Cx43 antibody (Abcam, #ab11370) it was applied according to a special protocol: perfusion of ACSF was stopped after the second SLE and Cx43 antibody was added to the chamber at a relatively high concentration (7.5 μg/ml, 1:100 dilution). After 10 min, the perfusion was restarted, and the antibody was washed out. In control measurements, the same protocol was applied without adding Cx43 antibody after perfusion was stopped. In these control measurements, SLE occurrence was not affected by the interruption of perfusion.

### Implantation of Rats for EEG Recording and Intracerebroventricular Treatment

Female WAG/Rij rats were used for *in vivo* experiments (11–12 months old, 195–210 g; breeding colony of WAG/Rij rats at ELTE Savaria University Centre, Szombathely, Hungary). Isoflurane-air mixture (2.0–2.5%) anesthesia was used for electrode implantation. Screw electrodes were implanted into the bone above two cortical areas of all rats for EEG recording (frontal cortex, AP: 2.0 mm and L: 2.1 mm; parietal cortex, AP: −6.5 mm and L: 2.1 mm) (Paxinos and Watson, 2007). A ground (screw) electrode was placed above the cerebellar cortex whereas the reference electrode (a stainless steel plate, 3 mm × 4 mm with one side insulated) was implanted under the skin and over the masseter muscle. The plate and electrodes were soldered to a 10-pin socket, which were glued to the skull by dentacrylate cement (Ivoclar, Liechtenstein). Lidocaine ointment (5%; EGIS, Hungary) was used for post-operative pain relief (Kovács et al., 2006).

One stainless steel guide cannula (27G) was inserted into the lateral ventricle (AP: −0.8 mm, L: 1.4 mm and V: 3.5 mm) to i.c.v. injection of TMA by infusion pump (WPI, Germany). The location of the cannula tip in the third ventricle was verified by the free outflow of cerebrospinal fluid (CSF) through the guide cannula. Patency of guide cannula was maintained with a sterile stainless steel dummy stylet (Kovács et al., 2011).

After implantations, all rats were allowed to recover for 2 weeks.

### *In vivo* Electrophysiology

Electroencephalogram (EEG) was recorded by a differential biological amplifier (Bioamp4, Supertech, Ltd., Pécs, Hungary). The amplifier was attached to a CED 1401 mkII (Cambridge Electronic Design, Ltd., United Kingdom) data capture and analysis device. The bandwidth of the EEG recording was 0.16 to 150 Hz and the sampling rate was 1 kHz (Kovács et al., 2014). As handling may evoke stress-induced changes in behavior for about 30 min, which can modify SWD number (Coenen and Van Luijtelaar, 2003; Kovács et al., 2015), evaluation of SWD parameters were carried out between 30 and 270 min of recording period between 3:00 and 7:00 PM. After the drug administration and the connection of rats to the biological amplifier, normal grooming and behavior were observed in all animals.

### *In vivo* Drug Application

To adapt the WAG/Rij rats to the experimental procedures, all animals were handled daily for 4 days, which adaptation period was started 2 weeks after surgery (for example rats were gently restrained by a towel and were connected to the biological amplifier).

Animals were separated into three groups. The first group of WAG/Rij rats (*n* = 8) was intraperitoneally (i.p.) injected by 0.5 ml/100 g body weight saline for 3 days (3-day control period) to establish average control SWD number and SWD time. On the 4th day, animals of group 1 were injected i.p. by 0.5 ml/100 g body weight saline containing CBX (100 mg/kg). The second group of animals (*n* = 5) received the i.c.v. TMA injection after the 3-day control period. The TMA solution (200 mM TMA in 5 μl aseptic ACSF/rat) was injected into the lateral ventricle through the guide cannula. At the beginning of the i.c.v. injection the animals were gently restrained by a towel. The dummy stylet was removed from the guide cannula and a stainless steel needle (which was connected to an infusion pump by a polyethylene tube) was inserted. The injection flow rate was 0.5 μl/min. The i.c.v. administration of 5 μl ACSF alone had no effect on SWD number (Kovács et al., 2011). The third group of animals (*n* = 7) were injected similar to animals of the first group by saline for 3 days (3-day control period) whereas they were injected i.p. by 0.5 ml/100 g body weight saline containing quinine (40 mg/kg) on the 4th day.

### Data Processing

The Clampfit (Axon Instruments) program was used to evaluate electrophysiological data. Recordings were analyzed after high pass filtering at 1 or 2 Hz. SLE onset was determined by the negative FP deflection and a high-frequency oscillation at the start of discharges. This is the paroxysmal initiation period, which is followed by the tonic and clonic periods of paroxysmal spike discharges (Lasztóczi et al., 2004). The interictal period was determined as the time from the end of a given SLE to the beginning of the next SLE. Being not fully developed, the first SLE in each slice (SLE0) was discarded from data evaluation. Data are shown as mean ± SEM and were analyzed with one-way analysis of variances (ANOVA, OriginPro 2018). Statistical significance was considered at *p* < 0.05.

*In vivo* EEG recordings were split into 30 min sections and were evaluated separately (Kovács et al., 2006). Spike-wave discharges can be characterized by 7–11 Hz discharge frequency within SWDs, 1–50 s duration and 0.2–1.0 mV amplitude (Coenen and Van Luijtelaar, 2003). Moreover, SWDs contain a train of asymmetric spikes and slow waves starting and ending with sharp spikes and the average amplitude of SWDs is at least twice as high as the basal EEG activity. These features of SWDs were used for automated separation of SWDs in the EEG files, confirmed by manual supervision. All results are expressed as means ± SEM and data analysis was performed similar to the *in vitro* results.

## Results

### Astrocytic Gap Junction Opening Increases Incidence of Recurrent Seizure-Like Events *in vitro*

**FIGURE 1:**
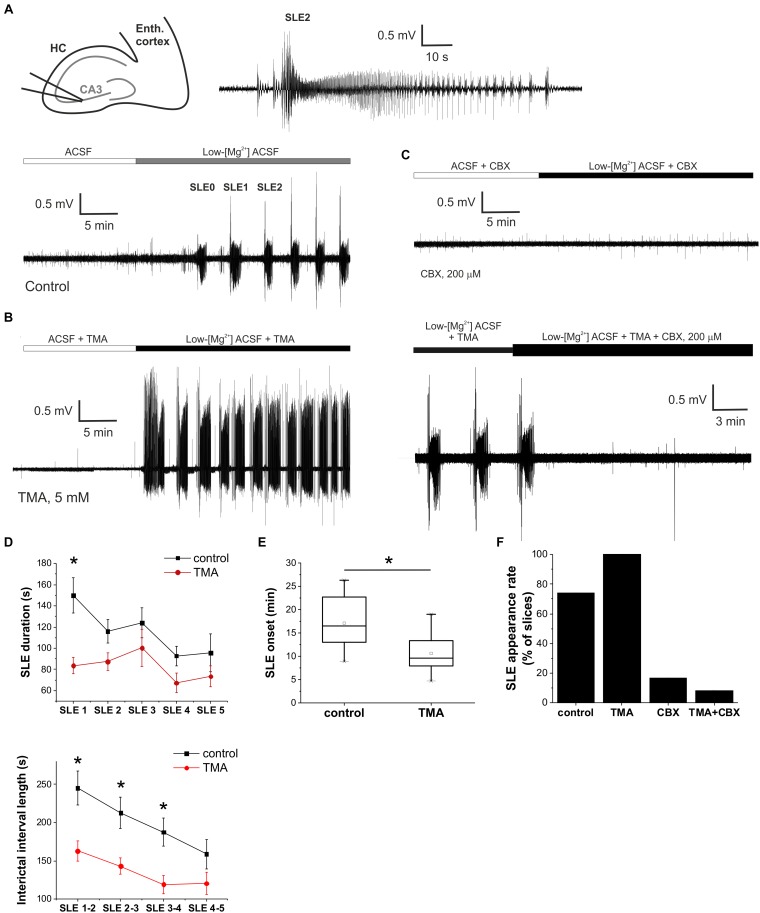
Gap junction activation enhances, gap junction inhibition suppresses epileptiform activity *in vitro* in the low-[Mg^2+^] TLE model. **(A)** Extracellular field potential (FP) recording of recurrent seizure-like events (SLEs) induced by low-[Mg^2+^] ACSF in the CA3 region of a hippocampal slice (top left). SLE2 is shown in higher temporal resolution (top right). **(B)** Extracellular FP recording of SLEs in the presence of 5 mM trimethylamine (TMA). **(C)** Extracellular FP recording in the presence of 200 μM carbenoxolone (CBX). CBX was applied either before the application of the low-[Mg^2+^] medium (top) or following the fully developed SLEs in the presence of 5 mM TMA (bottom). **(D)** Average duration of SLEs (top) and interictal periods (bottom) in control conditions (black) and in the presence of 5 mM TMA (red). Asterisks denote significant differences (*p* < 0.05) between control conditions and TMA application. **(E)** Onset of first SLE in control conditions and in the presence of 5 mM TMA. Application of TMA significantly decreases SLE onset. **(F)** Percentage of slices in which low-[Mg^2+^] ACSF induced SLEs.

We monitored the contribution of astrocytic gap junction channels to *in vitro* epileptiform activity in the CA3 region of juvenile rat acute hippocampal slices in the low-Mg^2+^ *in vitro* epilepsy model. Epileptiform discharges were induced by applying nominally Mg^2+^ free ACSF. Recurrent SLEs have been characterized by paroxysmal depolarization shifts before the onset of SLEs appeared in the presence of low-Mg^2+^ ACSF (Figure 1A). In control conditions (low-Mg^2+^ ACSF) recurrent SLEs appeared in 27 of 37 slices (73%). The average onset of the first SLE was 17.0 ± 1.0 min. Under these circumstances the average duration of SLEs were decreasing (SLE1: 149.7 ± 16.7 s, SLE2: 116 ± 11.3 s; SLE3: 124.1 ± 14.1 s; SLE4: 92.8 ± 9.2 s, SLE5: 95.7 ± 17.9 s). The interictal intervals during these recurrent SLEs were gradually declined (SLE 1–2: 245.2 ± 21.9 s; SLE 2–3: 212.7 ± 20.2 s; SLE 3–4: 187.4 ± 18.2 s; SLE 4–5: 158.7 ± 19.1 s) indicating a growing incidence of seizure propagation (Figures 1A,D).

We first explored the effect of the astrocytic gap junction opener TMA on the onset, duration and interictal interval of SLEs. The physiological activity (in normal ACSF) was not affected by the gap junction opener TMA (5 mM) (Figure 1B). However, TMA significantly decreased the onset and interictal interval of SLEs (Figure 1D). Interictal intervals significantly decreased to 162.9 ± 13.1 s (SLE 1–2); 143.1 ± 10.6 s (SLE 2–3); 119.1 ± 11.8 s (SLE 3–4) (*p* < 0.05 compared to the control period; *n* = 15). The onset of SLEs also diminished from 17.0 ± 1.0 to 10.6 ± 1.0 min compared to control condition (Figure 1E), increasing the incidence of recurrent seizure-like activity *in vitro.* These findings suggest that TMA application has a pro-epileptic effect.

However, the duration of SLEs also decreased to 83.6 ± 7.7 s (SLE1); 87.4 ± 8.1 s (SLE2); 100.3 ± 17.5 s (SLE3); 67.2 ± 9.2 s (SLE4); 73.4 ± 9.8 s (SLE5) (*p* = 0.01 for SLE1, *p* > 0.05 for ongoing SLEs compared to the control period; *n* = 15), showing that TMA also has a slight antiepileptic effect.

### Gap Junction Inhibition by Carbenoxolone Suppresses Seizure-Like Events *in vitro*

Next, we aimed to explore the effect of astrocytic gap junction inhibition on SLEs. We applied the gap junction blocker CBX (200 μM). In accordance with our previous reports (Kékesi et al., 2015), we observed that CBX treatment either inhibited or completely prevented SLE formation. SLE appearance was observed in only 2 of 12 slices (17%) (Figures 1C,F). Even in these two slices where CBX did not completely prevent SLE generation, it still reduced SLE duration. In the presence of CBX the average duration of the first SLE was 61 ± 18 s compared to 149.7 ± 16.7 s in control condition.

Unfortunately, neither CBX nor TMA are specific to gap junctions. TMA may induce intracellular calcium mobilization from ER calcium stores (Willoughby et al., 2001) or, as a weak base, may be responsible for the intracellular alkalization-induced calcium rise in a dose-dependent manner, a mechanism that is behind the gap junction opening effect as well (Willoughby et al., 2001; Nassiri-Asl et al., 2008; Medina-Ceja and Ventura-Mejía, 2010). CBX also has considerable influence on synaptic transmission with direct action on neuronal membrane conductances, as it reduces AMPA, NMDA, and GABA_A_ receptor-mediated excitatory and inhibitory post-synaptic currents (Vessey et al., 2004; Tovar et al., 2009; Connors, 2012) and inhibits voltage-gated Ca^2+^ channels (Rouach et al., 2003; Vessey et al., 2004). To confirm that their observed effects on SLEs was mediated by gap junctional interactions, we examined whether CBX can antagonize TMA-induced responses. In doing so we confirmed that 200 μM CBX in combination with 5 mM TMA suppressed the appearance of SLEs in 11 of 12 slices (92%) (Figure 1F). CBX was also found effective in reversing the pro-epileptic effect of TMA when it was applied after two fully developed SLEs (Figure 1C). *In situ* application of CBX typically resulted in the appearance of a single SLE followed by complete blockade of epileptiform activity in 7 of 12 slices (Figure 1C), suggesting that CBX and TMA shares a common target through which they affect epileptic activity.

Furthermore, we compared the incidence of SLEs in the control condition and in the three different treatments. When TMA was added, the incidence of recurrent SLEs increased to 100% (*n* = 15) compared to the control periods where it was only 72.97% (*n* = 37). In the remaining two treatments SLE formation was significantly declined. In the presence of CBX only 16.6% of slices generated SLEs (*n* = 12), while simultaneously applying TMA and CBX induced SLEs only in 8.3% of slices (*n* = 12) (Figure 1F).

### Astrocytic Connexin Channels Are Responsible for the Modulation of Seizure-Like Events

**FIGURE 2:**
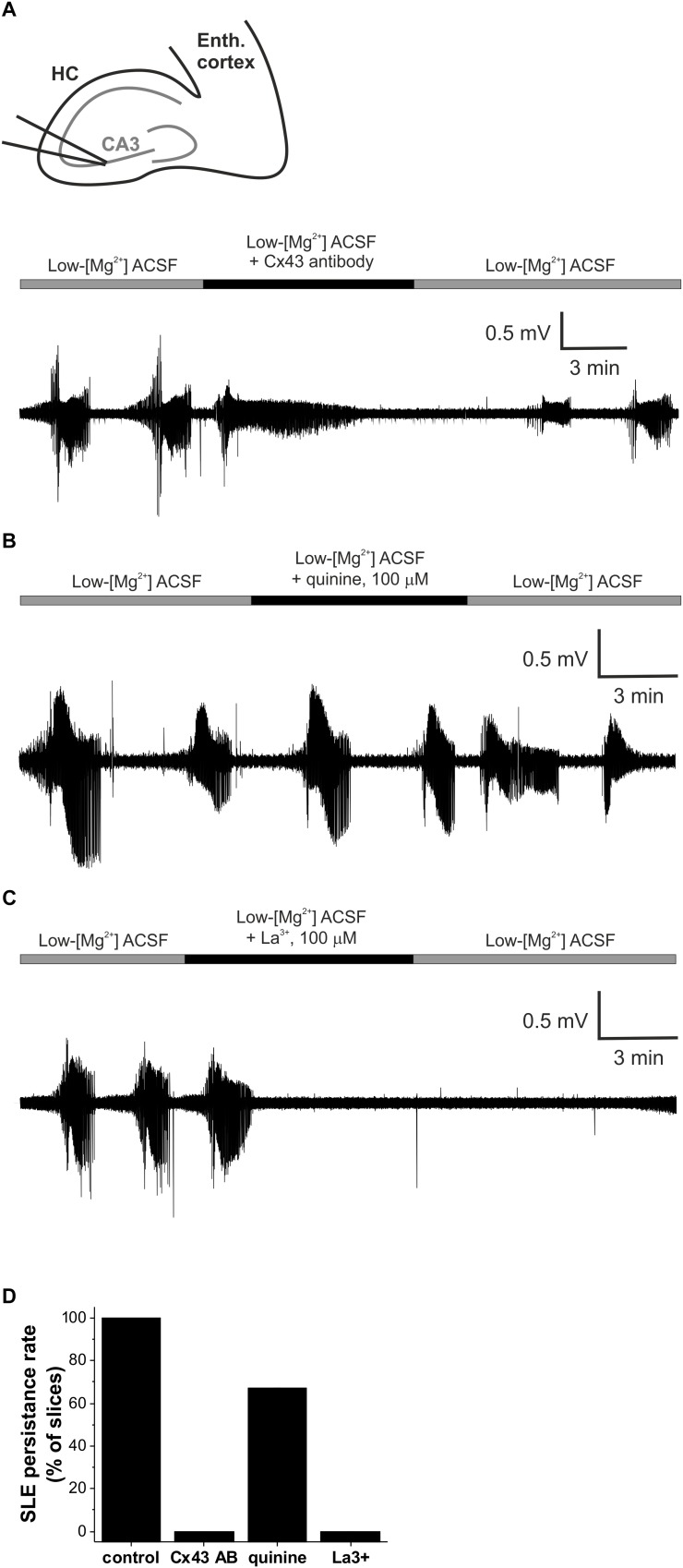
Gap junctional control over epileptiform activity is mediated specifically by the astrocytic gap junctions *in vitro* in the low-[Mg^2+^] TLE model. **(A)** Representative extracellular field potential (FP) recording of recurrent seizure-like events (SLEs) under control conditions and in the presence of a Cx43-specific antibody specifically blocking astrocytic connexin channels in the CA3 region of a hippocampal slice. **(B)** Representative extracellular FP recording of recurrent SLEs under control conditions and in the presence of the Cx36-specific quinine specifically blocking neuronal connexin channels in the CA3 region of a hippocampal slice. **(C)** Representative extracellular FP recording of recurrent SLEs under control conditions and in the presence of the hemichannel-specific La(NO_3_)_3_ in the CA3 region of a hippocampal slice. **(D)** Percentage of slices in which SLEs persisted in the presence of gap junction inhibitors applied after two fully developed SLEs in low-[Mg^2+^] ACSF. SLEs were considered to be persisted if at least two SLEs appeared during the application of the given inhibitor.

Although we confirmed that TMA and CBX share a common target in regard to their modulatory activity on SLEs, the molecular identity of this target was not fully resolved. First, to differentiate between the neuronal and astrocytic connexins, we compared the effects of a Cx43- and a Cx36-specific inhibitor. To specifically block the astrocytic Cx43 subtype, we used an antibody that was raised against the gating peptide segment of Cx43 (Sosinsky et al., 2007), which we previously demonstrated to block Cx43 function (Molnár et al., 2011; Kékesi et al., 2015; Szabó et al., 2017). The specific Cx43 antibody completely eliminated SLEs in 5 of 5 slices – although a residual, non-epileptiform activity was usually observed (Figure 2A).

To further confirm the connexin subtype-specificity of the gap junction-related anti-epileptic effect, we also investigated the contribution of the neuronal Cx36 subtype, which is known to be involved in neuronal synchronization (Allen et al., 2011). Cx36 was specifically inhibited by 100 μM quinine. In contrast to Cx43 inhibition, however, application of quinine did not eliminate the appearance of seizures (Figure 2B). SLEs continued to be generated in 7 of 11 slices in the presence of quinine (Figure 2D), demonstrating the major role of astrocytic Cx43 in the contribution of connexin isoforms to epileptiform activity.

Finally, we explored whether connexin hemichannels may play a role in the modulatory effects of gap junction proteins on epileptiform activity. To achieve this, we applied lanthanum (La^3+^), which is supposed to selectively block hemichannel function (Contreras et al., 2002; Braet et al., 2003; Anselmi et al., 2008). SLEs have been completely abolished in 5 of 5 slices in the presence of 100 μM La^3+^ (Figures 2C,D), demonstrating that hemichannels are involved in the regulation of epileptiform activity. It is to note, however, that despite the indisputable efficiency of La^3+^ on hemichannels SLEs, only a small number of direct studies (Contreras et al., 2002; Anselmi et al., 2008) explored its effect on full gap junctions and no experimental data is available in complex tissues, like acute brain slices or *in vivo* brain. Therefore, the inhibition of SLEs by La^3+^ does not exclude the involvement of full gap junctions.

### Gap Junctions Have Distinct Effect on Absence Epileptic Activity

The gap junction blocker CBX showed anticonvulsant characteristics as opposed to the convulsive attributes of the gap junction opener TMA in the *in vitro* TLE model. To explore whether this seemingly pro-epileptic gap junction activity is a general hallmark of epilepsy or it is rather a feature of only convulsive seizures, we determined the effects of gap junction blockade and opening in the genetically epileptic WAG/Rij rats, a model of the non-convulsive human absence epilepsy.

**FIGURE 3:**
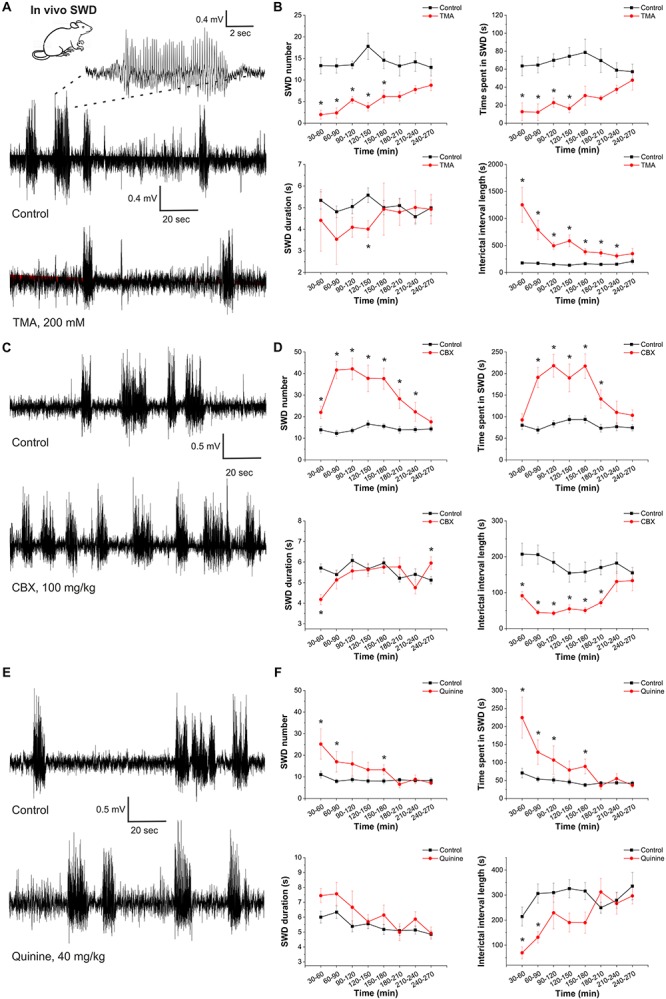
Gap junction activation suppresses, gap junction inhibition enhances the appearance of spike-and-wave discharges in the *in vivo* absence epilepsy model WAG/Rij rat. **(A)** EEG recordings of WAG/Rij rats after i.p. saline injection (control) and TMA administration (i.c.v. 200 mM TMA in 5 μl aseptic ACSF/rat) (left). A typical SWD is shown in higher resolution (above). **(B)** Effect of TMA on SWD number, total SWD time (time spent in SWD), average SWD duration and length of interictal interval (control: black; treated with TMA: red) (*n* = 5 animals). **(C)** EEG recordings of WAG/Rij rats after i.p. saline injection (control) and CBX administration (i.p. 100 mg/kg). **(D)** Effect of CBX on SWD number, total SWD time (time spent in SWD), average SWD duration and length of interictal interval (control: black; treated with CBX: red) (*n* = 8 animals). **(E)** EEG recordings of WAG/Rij rats after i.p. saline injection (control) and quinine administration (i.p. 40 mg/kg). **(F)** Effect of quinine on SWD number, total SWD time (time spent in SWD), average SWD duration and length of interictal interval (control: black; treated with quinine: red) (*n* = 7 animals).

In WAG/Rij rats, intracerebroventricular injection of TMA (200 mM) significantly decreased the SWD number in all 30-min segments compared to controls (Figures 3A,B and Table 1). Parallel with decreased SWD number, the length of interictal interval significantly increased (Figure 3B). As average SWD duration did not change after TMA administration, the total time of SWDs decreased in parallel with the reduction in SWD number (Figure 3B).

**TABLE 1:** Synchronous spike-wave discharge (SWD) parameters in WAG/Rij rats (*n* = 5) in control conditions and after i.p. administration of 200 mM trimethylamine (TMA).

Time (min)	SWD number		Time spent in SWD (s)		SWD duration (s)		Interictal interval length (s)	
	Control	TMA	Control	TMA	Control	TMA	Control	TMA
30–60	13.33±1.90	2.00±1.05	63.67±10.91	12.81±9.83	5.34±0.40	4.41±1.43	177.41±26.57	1251.99±323.34
60–90	13.27±1.66	2.40±1.21	64.64±8.71	12.24±9.27	4.81±0.26	3.54±1.16	171.55±30.58	788.83±178.72
90–120	13.53±0.87	5.40±0.81	69.95±7.19	22.90±5.24	5.05±0.33	4.09±0.46	148.74±9.19	495.46±68.40
120–150	17.80±3.04	3.80±0.97	74.42±9.63	16.42±4.82	5.58±0.34	4.01±0.39	135.10±20.82	585.00±109.46
150–180	14.60±1.92	6.20±1.50	78.70±14.67	30.70±10.57	5.00±0.34	4.93±1.21	163.36±24.63	384.28±57.93
180–210	13.27±2.29	6.20±1.07	69.66±13.17	27.71±2.85	5.09±0.34	4.79±0.61	150.43±18.00	361.95±65.15
210–240	14.20±2.22	7.80±0.92	58.99±8.16	37.65±4.65	4.58±0.30	5.01±0.78	154.00±17.98	306.93±52.46
240–270	12.93±1.99	8.80±1.25	57.25±8.44	47.86±11.05	4.99±0.37	4.93±0.68	206.43±42.96	348.79±96.80

In contrast to TMA, i.p. administration of 100 mg/kg CBX significantly increased the SWD number and total time of SWDs. The length of interictal intervals significantly decreased (Figures 3C,D and Table 2).

**TABLE 2:** Synchronous spike-wave discharge (SWD) parameters in WAG/Rij rats (*n* = 8) in control conditions and after i.c.v. administration of 100 mg/kg carbenoxolone (CBX).

Time (min)	SWD number		Time spent in SWD (s)		SWD duration (s)		Interictal interval length (s)	
	Control	CBX	Control	CBX	Control	CBX	Control	CBX
30–60	13.92±1.59	22.00±2.85	80.23±9.48	92.67±14.23	5.71±0.20	4.18±0.25	207.12±30.89	91.21±11.46
60–90	12.29±1.40	41.63±4.04	69.15±7.67	191.10±23.59	5.39±0.22	5.13±0.44	205.79±26.71	44.92±6.39
90–120	13.58±1.16	42.13±4.98	83.46±7.62	218.08±26.33	6.08±0.29	5.57±0.39	184.46±26.98	42.55±6.43
120–150	16.58±1.56	37.75±6.12	93.51±9.39	189.8±32.01	5.66±0.23	5.63±0.32	154.48±25.33	54.82±11.08
150–180	15.58±1.16	37.63±4.87	93.77±8.31	217.11±28.40	5.96±0.24	5.76±0.31	157.46±27.48	50.23±8.16
180–210	13.92±1.57	28.25±4.49	73.31±8.81	141.07±21.19	5.21±0.19	5.76±0.46	170.03±20.25	72.04±10.10
210–240	14.04±1.30	22.25±4.37	76.90±9.31	109.96±26.25	5.40±0.27	4.76±0.30	182.25±28.23	130.72±28.21
240–270	14.33±1.16	17.63±2.20	74.48±7.14	103.27±12.57	5.12±0.18	5.95±0.31	155.00±15.13	133.39±28.14

In addition, we investigated the connexin isoform-specificity in the *in vivo* absence epilepsy model as well. Similar to CBX, i.p. administration of the Cx36-specific inhibitor quinine (40 mg/kg) also increased the number and total time of SWDs and decreased the length of the interictal intervals (Figures 3E,F and Table 3). The magnitude of these changes, however, were inferior to CBX treatment. Compared to the average 132% increase in the SWD number in the first 4 h following CBX treatment, quinine induced only 51% increase in the same period. Since the Cx36-specific inhibitor quinine mimicked CBX effect only to a minor extent, we conclude that the majority of gap junction-related modulation of seizure activity is performed through the astrocytic Cx43 isoform.

**TABLE 3:** Synchronous spike-wave discharge parameters in WAG/Rij rats (*n* = 7) in control conditions and after i.p. administration of 40 mg/kg quinine.

Time (min)	SWD number		Time spent in SWD (s)		SWD duration (s)		Interictal interval length (s)	
	Control	Quinine	Control	Quinine	Control	Quinine	Control	Quinine
30–60	11.05±1.64	25.17±7.03	70.79±13.26	224.47±57.15	6.00±0.40	7.45±0.47	214.23±37.68	69.38±24.02
60–90	7.95±1.00	17.00±4.73	53.47±7.16	128.63±33.94	6.34±0.42	7.57±0.77	306.30±38.26	131.46±22.60
90–120	8.71±1.14	16.00±5.60	51.14±8.46	106.61±39.73	5.39±0.37	6.66±1.09	309.84±37.89	229.18±64.44
120–150	8.10±1.06	13.29±3.52	45.60±6.96	79.04±24.82	5.56±0.31	5.69±0.47	325.82±36.12	190.37±37.45
150–180	8.05±1.23	13.29±2.31	37.39±5.12	88.87±20.49	5.18±0.33	6.14±0.67	315.98±33.56	189.73±42.43
180–210	8.62±0.85	6.57±1.59	42.69±5.45	35.34±9.62	5.09±0.31	4.99±0.57	249.53±21.85	312.32±53.84
210–240	8.29±0.75	8.86±2.02	43.50±5.11	54.92±14.08	5.14±0.27	5.87±0.50	279.50±31.61	266.60±42.83
240–270	8.29±1.16	7.14±0.80	42.23±6.66	36.56±6.34	4.85±0.25	4.92±0.42	335.88±54.46	296.79±32.31

Overall, although a common gap junctional target may be conjectured in the *in vitro* and *in vivo* models, opposite effects of TMA and CBX demonstrated on absence epileptic activity in WAG/Rij rats compared to the *in vitro* TLE model suggests different role of gap junctions in convulsive and non-convulsive epilepsies.

## Discussion

We have observed that in the *in vitro* low-[Mg^2+^] model and in the WAG/Rij rats *in vivo*, gap junction openers and blockers showed distinct effects on epileptiform activity. In the low-[Mg^2+^] model, which is a model of the convulsive TLE (Mody et al., 1987) gap junction opening by TMA significantly increased the prevalence of SLEs, while the gap junction inhibitor CBX almost completely prevented the appearance of SLEs. Therefore, gap junction activity appears to be pro-epileptic in this model. Pharmacological dissection of the molecular target identified that the major contributor of the gap junctional control over epileptiform activity is the astrocytic Cx43. Although the *in vitro* and *in vivo* environments can be attributed to the different effects, it is of outmost importance that TMA and CBX showed pro- and anticonvulsant properties, respectively, also in *in vivo* TLE models (Gajda et al., 2003, 2006). Also, in agreement with our *in vitro* results, the supposedly Cx36 specific quinine only slightly affected epileptogenesis in the same TLE model (Gajda et al., 2005). Therefore, the differential outcomes in the two epilepsy models may not be attributed to the *in vitro* vs. *in vivo* environment, but rather to the different epilepsy types they model, i.e., the convulsive TLE and the non-convulsive absence epilepsy.

### Proposed Model for Differential Role of Astrocytic Gap Junctions in Seizure Spreading

The experimental findings outlined above clearly demonstrate that astrocytic hemichannel activity is indispensable for the appearance of recurrent epileptiform activity in the low-[Mg^2+^] model, since CBX, the Cx43-specific antibody and the hemichannel blocker La^3+^ could completely eliminate SLEs. In the absence epilepsy model WAG/Rij rats, gap junctions appeared to play an opposite role, they had to be activated to inhibit (but not completely block) SWDs. These results reflect the dual role of gap junctions in epilepsy. Intercellular gap junctions play a pivotal role in K^+^ buffering and Glu clearance, since they contribute to the spatial distribution of these excitatory agents (Seifert et al., 2012). Therefore, intercellular gap junctions primarily play an anti-epileptic role. On the other hand, they also promote synchronization (Gigout et al., 2006) by which they exert pro-epileptic effect. Importantly, hemichannels mostly contribute to the pro-epileptic action by releasing Glu and the NMDA receptor co-agonist D-serine (Abudara et al., 2018). The final outcome of gap junction blockade or activation, therefore, depends on the relative weights of the anti-epileptic and pro-epileptic pathways.

**FIGURE 4:**
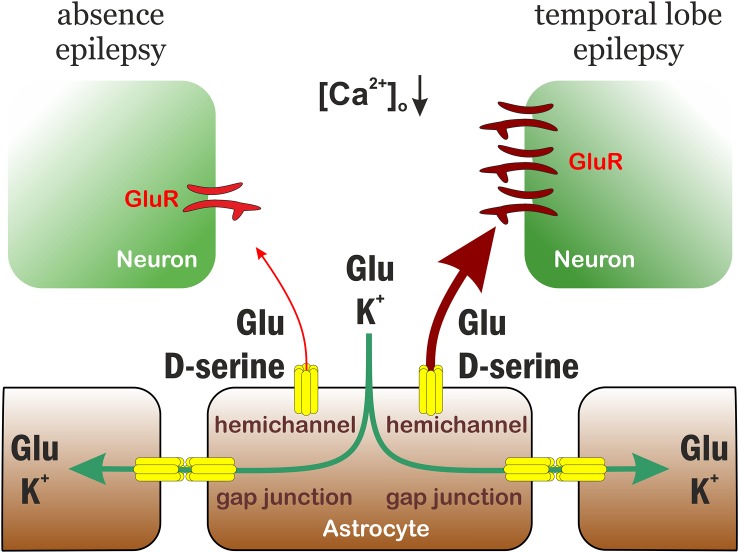
Proposed model for differential role of astrocytic gap junctions in seizure spreading in temporal lobe and absence epilepsies. The astrocytic connexin Cx43 has a dual role in temporal lobe and absence epilepsies. It performs an anti-epileptic action by spatial distribution of Glu and K^+^. On the other hand, connexin hemichannels release Glu and D-serine that increases neuronal excitation, therefore contributes to seizure generation and maintenance. The weight of the two pathways, however, are distinct in temporal lobe and absence epilepsies, because the expression level of neuronal (extrasynaptic) Glu receptors is significantly higher in temporal lobe epilepsy. Consequently, the final outcome of connexin activation is an anti-epileptic effect in absence epilepsy, but a pro-epileptic effect in temporal lobe epilepsy.

We propose that the differential effects observed in the TLE and absence epilepsy models can be attributed to the distinct weights of anti-epileptic and pro-epileptic gap junction-mediated routes (Figure 4). There is no data available about specific changes in the capacity or speed of Glu and K^+^ clearance in TLE and absence epilepsy. There is, however, a significant disparity regarding the consequences of hemichannel-mediated release. In response to the reduced extracellular [Ca^2+^] during seizures (Heinemann et al., 1986; Kovács et al., 2000), hemichannels increase opening probability (Stout and Charles, 2003) and release various substances, including Glu, D-serine, and ATP (Xing et al., 2019). Amongst these, the extent of response generated by Glu and D-serine is markedly different in TLE and absence epilepsy. One of the hallmarks of absence epilepsy is the significantly reduced expression of Glu receptors. Both AMPA and NMDA receptors (van de Bovenkamp-Janssen et al., 2006), especially the major extrasynaptic NR2B subunits (Karimzadeh et al., 2013) display a significantly lower expression level in WAG/Rij rats compared to non-epileptic strains. In contrast, Glu receptor expression was found to be even higher in TLE patients than in non-epileptic control (Mathern et al., 1997). Therefore, Glu and D-serine release results in significantly higher excitatory drive in TLE than in absence epilepsy that have the potential to counteract the anti-epileptic action of Glu and K^+^ clearance.

Moreover, the differential effect of gap junction inhibitors on epileptiform activity may also be attributed to regional differences in connexin composition. The primary brain region affected by TLE is the hippocampus, while absence epilepsy is mostly attributed to the thalamo-cortical network. While hippocampal astrocytes are dominated by the Cx43 isoform, the major connexin in thalamic astrocytes is Cx30 and many thalamic astrocytes do not even express Cx43 (Griemsmann et al., 2015). Cx43 expression have been reported to increase after seizures by several groups (Samoilova et al., 2003; Mylvaganam et al., 2010). In contrast, Cx30 mRNA is even downregulated during generalized seizures (Akbarpour et al., 2012). Consequently, connexin channel-mediated pathways may produce stronger pro-epileptic responses in TLE than in absence epilepsy.

Overall, our findings suggest that astrocytic and/or neuronal gap junctions have a different role in the modulation of absence epileptic activity compared to TLE. Further studies are needed to reveal the specific action mechanisms linked to astrocytic and/or neuronal gap junctions on different types of epileptic activities.

## Ethics Statement

Animals were kept and used in accordance with standard ethical guidelines and approved by the local Animal Care Committee, the Government Office for Pest County (Reference Nos. PEI/001/3671-4/2015 and PE/EA/3840-4/2016), the Hungarian Act of Animal Care and Experimentation (1998, XXVIII, section 243), European Communities Council Directive 24 November, 1986 (86/609/EEC) and EU Directive 2010/63/EU on the use and treatment of animals in experimental laboratories. The experiments on WAG/Rij rats were approved by the Animal Care and Experimentation Committee of the Eötvös Loránd University (Savaria University Centre) and National Scientific Ethical Committee on Animal Experimentation (Hungary) under License No. VA/ÉBNTF02/85-8/2016. All efforts were made to reduce animal suffering and the number of animals used.

## Author Contributions

RV performed the *in vitro* experiments and wrote the manuscript. MP performed the *in vitro* experiments. ZS analyzed the data. JK designed the experiments and wrote the manuscript. LH designed the experiments, analyzed the data, and wrote the manuscript. ZK performed the *in vivo* experiments and wrote the manuscript.

## Conflict of Interest Statement

The authors declare that the research was conducted in the absence of any commercial or financial relationships that could be construed as a potential conflict of interest.

## References

[B1] Abudara V. Retamal M. A. Del Rio R. Orellana J. A. (2018). Synaptic functions of hemichannels and pannexons: a double-edged sword. *Front. Mol. Neurosci.* 11:435. 10.3389/fnmol.2018.00435 30564096 PMC6288452

[B2] Akbarpour B. Sayyah M. Babapour V. Mahdian R. Beheshti S. Kamyab A. R. (2012). Expression of connexin 30 and connexin 32 in hippocampus of rat during epileptogenesis in a kindling model of epilepsy. *Neurosci. Bull.* 28 729–736. 10.1007/s12264-012-1279-6 23149765 PMC5561816

[B3] Allen K. Fuchs E. C. Jaschonek H. Bannerman D. M. Monyer H. (2011). Gap junctions between interneurons are required for normal spatial coding in the hippocampus and short-term spatial memory. *J. Neurosci.* 31 6542–6552. 10.1523/JNEUROSCI.6512-10.2011 21525295 PMC3160467

[B4] Anselmi F. Hernandez V. H. Crispino G. Seydel A. Ortolano S. Roper S. D. (2008). ATP release through connexin hemichannels and gap junction transfer of second messengers propagate Ca2+ signals across the inner ear. *Proc. Natl. Acad. Sci. U.S.A.* 105 18770–18775. 10.1073/pnas.0800793105 19047635 PMC2596208

[B5] Avoli M. (2014). Mechanisms of epileptiform synchronization in cortical neuronal networks. *Curr. Med. Chem.* 21 653–662. 24251567 10.2174/0929867320666131119151136 PMC4880466

[B6] Bedner P. Steinhäuser C. (2013). Neurochemistry international altered kir and gap junction channels in temporal lobe epilepsy. *Neurochem. Int.* 63 682–687. 10.1016/j.neuint.2013.01.011 23357483

[B7] Bennett M. V. L. Contreras J. E. Bukauskas F. F. Sáez J. C. (2003). New roles for astrocytes: gap junction hemichannels have something to communicate. *Trends Neurosci.* 26 610–617. 10.1016/j.tins.2003.09.008 14585601 PMC3694339

[B8] Berkovic S. F. Mulley J. C. Scheffer I. E. Petrou S. (2006). Human epilepsies: interaction of genetic and acquired factors. *Trends Neurosci.* 29 391–397. 10.1016/j.tins.2006.05.009 16769131

[B9] Beyer E. C. Berthoud V. M. (2018). Membrane channels formed by gap junction proteins. *Biochim. Biophys. Acta Biomembr.* 1860 1–4. 10.1016/j.bbamem.2017.10.021 29113817

[B10] Beyer E. C. Berthoud V. M. Herve C. Herve C. (2018). Gap junction gene and protein families: connexins, innexins, and pannexins. *Biochim. Biophys. Acta Biomembr.* 1860 5–8. 10.1016/j.bbamem.2017.05.016 28559187 PMC5704981

[B11] Binder D. K. Nagelhus E. A. Ottersen O. P. (2012). Aquaporin-4 and epilepsy. *Glia* 60 1203–1214. 10.1002/glia.22317 22378467

[B12] Braet K. Aspeslagh S. Vandamme W. Willecke K. Martin P. E. M. Evans W. H. (2003). Pharmacological sensitivity of ATP release triggered by photoliberation of inositol-1,4,5-trisphosphate and zero extracellular calcium in brain endothelial cells. *J. Cell. Physiol.* 197 205–213. 10.1002/jcp.10365 14502560

[B13] Coenen A. M. L. Van Luijtelaar E. L. J. M. (2003). Genetic animal models for absence epilepsy: a review of the WAG/Rij strain of rats. *Behav. Genet.* 33 635–655. 14574120 10.1023/a:1026179013847

[B14] Connors B. W. (2012). Tales of a dirty drug: carbenoxolone, gap junctions, and seizures. *Epilepsy Curr.* 12 66–68. 10.5698/1535-7511-12.2.66 22473546 PMC3316363

[B15] Contreras J. E. Sanchez H. A. Eugenin E. A. Speidel D. Theis M. Willecke K. (2002). Metabolic inhibition induces opening of unapposed connexin 43 gap junction hemichannels and reduces gap junctional communication in cortical astrocytes in culture. *Proc. Natl. Acad. Sci. U.S.A.* 99 495–500. 10.1073/pnas.012589799 11756680 PMC117588

[B16] Cotrina M. L. Lin J. H. Alves-Rodrigues A. Liu S. Li J. Azmi-Ghadimi H. (1998). Connexins regulate calcium signaling by controlling ATP release. *Proc. Natl. Acad. Sci. U.S.A.* 95 15735–15740. 9861039 10.1073/pnas.95.26.15735 PMC28113

[B17] Eadie M. J. (2012). Shortcomings in the current treatment of epilepsy. *Expert Rev. Neurother.* 12 1419–1427. 10.1586/ern.12.129 23237349

[B18] Eid T. Lee T. S. W. Patrylo P. Zaveri H. P. (2018). Astrocytes and glutamine synthetase in epileptogenesis. *J. Neurosci. Res.* 10.1002/jnr.24267 [Epub ahead of print]. 30022509 PMC6338538

[B19] Gajda Z. Gyengési E. Hermesz E. Said Ali K. Szente M. (2003). Involvement of gap junctions in the manifestation and control of the duration of seizures in rats in vivo. *Epilepsia* 44 1596–1600. 10.1111/j.0013-9580.2003.25803.x 14636335

[B20] Gajda Z. Hermesz E. Gyengési E. Szupera Z. Szente M. (2006). The functional significance of gap junction channels in the epileptogenicity and seizure susceptibility of juvenile rats. *Epilepsia* 47 1009–1022. 10.1111/j.1528-1167.2006.00573.x 16822247

[B21] Gajda Z. Szupera Z. Blazsó G. Szente M. (2005). Quinine, a blocker of neuronal cx36 channels, suppresses seizure activity in rat neocortex in vivo. *Epilepsia* 46 1581–1591. 10.1111/j.1528-1167.2005.00254.x 16190928

[B22] Gareri P. Condorelli D. Belluardo N. Citraro R. Barresi V. Trovato-Salinaro A. (2005). Antiabsence effects of carbenoxolone in two genetic animal models of absence epilepsy (WAG/Rij rats and lh/lh mice). *Neuropharmacology* 49 551–563. 10.1016/j.neuropharm.2005.04.012 15936783

[B23] Gigout S. Louvel J. Kawasaki H. D’Antuono M. Armand V. Kurcewicz I. (2006). Effects of gap junction blockers on human neocortical synchronization. *Neurobiol. Dis.* 22 496–508. 10.1016/j.nbd.2005.12.011 16478664

[B24] Griemsmann S. Höft S. P. Bedner P. Zhang J. von Staden E. Beinhauer A. (2015). Characterization of panglial gap junction networks in the thalamus, neocortex, and hippocampus reveals a unique population of glial cells. *Cereb. Cortex* 25 3420–3433. 10.1093/cercor/bhu157 25037920 PMC4585496

[B25] Guthrie P. B. Knappenberger J. Segal M. Bennett M. V. L. Charles A. C. Kater S. B. (1999). ATP released from astrocytes mediates glial calcium waves. *J. Neurosci.* 19 520–528. 9880572 10.1523/JNEUROSCI.19-02-00520.1999 PMC6782195

[B26] Heinemann U. Konnerth A. Pumain R. Wadman W. J. (1986). Extracellular calcium and potassium concentration changes in chronic epileptic brain tissue. *Adv. Neurol.* 44 641–661. 3518350

[B27] Héja L. (2014). Astrocytic target mechanisms in epilepsy. *Curr. Med. Chem.* 21 755–763. 24251560 10.2174/0929867320666131119160445

[B28] Héja L. Barabás P. Nyitrai G. Kékesi K. A. K. A. Lasztóczi B. Toke O. (2009). Glutamate uptake triggers transporter-mediated GABA release from astrocytes. *PLoS One* 4:e7153. 10.1371/journal.pone.0007153 19777062 PMC2744931

[B29] Héja L. Nyitrai G. Kékesi O. Dobolyi A. Szabó P. Fiáth R. (2012). Astrocytes convert network excitation to tonic inhibition of neurons. *BMC Biol.* 10:26. 10.1186/1741-7007-10-26 22420899 PMC3342137

[B30] Hubbard J. A. Hsu M. S. Seldin M. M. Binder D. K. (2015). Expression of the astrocyte water channel aquaporin-4 in the mouse brain. *ASN Neuro.* 7:175909141560548. 10.1177/1759091415605486 26489685 PMC4623559

[B31] Karimzadeh F. Soleimani M. Mehdizadeh M. Jafarian M. Mohamadpour M. Kazemi H. (2013). Diminution of the NMDA receptor NR2B subunit in cortical and subcortical areas of WAG/Rij rats. *Synapse* 67 839–846. 10.1002/syn.21687 23754322

[B32] Kékesi O. Ioja E. Szabó Z. Kardos J. Héja L. (2015). Recurrent seizure-like events are associated with coupled astroglial synchronization. *Front. Cell. Neurosci.* 9:215. 10.3389/fncel.2015.00215 26150770 PMC4471369

[B33] Kofuji P. Newman E. A. (2004). Potassium buffering in the central nervous system. *Neuroscience* 129 1045–1056. 10.1016/j.neuroscience.2004.06.008 15561419 PMC2322935

[B34] Kohmann D. Lüttjohann A. Seidenbecher T. Coulon P. Pape H.-C. (2016). Short-term depression of gap junctional coupling in reticular thalamic neurons of absence epileptic rats. *J. Physiol.* 594 5695–5710. 10.1113/JP271811 26940972 PMC5043037

[B35] Kovács Z. Kékesi K. A. Dobolyi Á. Lakatos R. Juhász G. (2015). Absence epileptic activity changing effects of non-adenosine nucleoside inosine, guanosine and uridine in wistar albino glaxo rijswijk rats. *Neuroscience* 300 593–608. 10.1016/j.neuroscience.2015.05.054 26037802

[B36] Kovács R. Szilágyi N. Barabás P. Heinemann U. Kardos J. (2000). Low-[Mg2+]-induced Ca2+ fluctuations in organotypic hippocampal slice cultures. *Neuroreport* 11 2107–2111. 10923653 10.1097/00001756-200007140-00010

[B37] Kovács Z. Czurkó A. Kékesi K. A. Juhász G. (2011). Intracerebroventricularly administered lipopolysaccharide enhances spike-wave discharges in freely moving WAG/Rij rats. *Brain Res. Bull.* 85 410–416. 10.1016/j.brainresbull.2011.05.003 21619914

[B38] Kovács Z. Dobolyi A. Juhász G. Kékesi K. A. (2014). Lipopolysaccharide induced increase in seizure activity in two animal models of absence epilepsy WAG/Rij and GAERS rats and long evans rats. *Brain Res. Bull.* 104 7–18. 10.1016/j.brainresbull.2014.03.003 24704320

[B39] Kovács Z. Kékesi K. A. Szilágyi N. Abrahám I. Székács D. Király N. (2006). Facilitation of spike-wave discharge activity by lipopolysaccharides in wistar albino Glaxo/rijswijk rats. *Neuroscience* 140 731–742. 10.1016/j.neuroscience.2006.02.023 16616432

[B40] Lasztóczi B. Antal K. Nyikos L. Emri Z. Kardos J. (2004). High-frequency synaptic input contributes to seizure initiation in the low-[Mg2+] model of epilepsy. *Eur. J. Neurosci.* 19 1361–1372. 10.1111/j.1460-9568.2004.03231.x 15016094

[B41] Mathern G. W. Pretorius J. K. Kornblum H. I. Mendoza D. Lozada A. Leite J. P. (1997). Human hippocampal AMPA and NMDA mRNA levels in temporal lobe epilepsy patients. *Brain* 120(Pt 11), 1937–1959. 9397013 10.1093/brain/120.11.1937

[B42] Medina-Ceja L. Ventura-Mejía C. (2010). Differential effects of trimethylamine and quinine on seizures induced by 4-aminopyridine administration in the entorhinal cortex of vigilant rats. *Seizure* 19 507–513. 10.1016/j.seizure.2010.07.009 20685138

[B43] Mody I. Lambert J. D. Heinemann U. (1987). Low extracellular magnesium induces epileptiform activity and spreading depression in rat hippocampal slices. *J. Neurophysiol.* 57 869–888. 3031235 10.1152/jn.1987.57.3.869

[B44] Molnár T. Dobolyi A. Nyitrai G. Barabás P. Héja L. Emri Z. (2011). Calcium signals in the nucleus accumbens: activation of astrocytes by ATP and succinate. *BMC Neurosci.* 12:96. 10.1186/1471-2202-12-96 21967230 PMC3199278

[B45] Mukai T. Kinboshi M. Nagao Y. Shimizu S. Ono A. Sakagami Y. (2018). Antiepileptic drugs elevate astrocytic Kir4.1 expression in the rat limbic region. *Front. Pharmacol.* 9:845. 10.3389/fphar.2018.00845 30127740 PMC6088221

[B46] Mulley J. C. Scheffer I. E. Harkin L. A. Berkovic S. F. Dibbens L. M. (2005). Susceptibility genes for complex epilepsy. *Hum. Mol. Genet.* 14 R243–R249. 10.1093/hmg/ddi355 16244322

[B47] Mylvaganam S. Zhang L. Wu C. Zhang Z. J. Samoilova M. Eubanks J. (2010). Hippocampal seizures alter the expression of the pannexin and connexin transcriptome. *J. Neurochem.* 112 92–102. 10.1111/j.1471-4159.2009.06431.x 19840216

[B48] Nassiri-Asl M. Zamansoltani F. Zangivand A. A. (2008). The inhibitory effect of trimethylamine on the anticonvulsant activities of quinine in the pentylenetetrazole model in rats. *Prog. Neuropsychopharmacol. Biol. Psychiatry* 32 1496–1500. 10.1016/j.pnpbp.2008.05.007 18556104

[B49] Paxinos G. Watson C. (2007). *The Rat Brain in Stereotaxic Coordinates.* Amsterdam: Elsevier. 10.1016/0165-0270(80)90021-7 6110810

[B50] Robin L. M. Oliveira da Cruz J. F. Langlais V. C. Martin-Fernandez M. Metna-Laurent M. Busquets-Garcia A. (2018). Astroglial cb1 receptors determine synaptic d-serine availability to enable recognition memory. *Neuron* 98 935–944.e5. 10.1016/j.neuron.2018.04.034 29779943

[B51] Rouach N. Koulakoff A. Abudara V. Willecke K. Giaume C. (2008). Astroglial metabolic networks sustain hippocampal synaptic transmission. *Science* 322 1551–1555. 10.1126/science.1164022 19056987

[B52] Rouach N. Segal M. Koulakoff A. Giaume C. Avignone E. (2003). Carbenoxolone blockade of neuronal network activity in culture is not mediated by an action of gap junctions. *J. Physiol.* 553 729–745. 10.1113/jphysiol.2003.053439 14514879 PMC2343628

[B53] Samoilova M. Li J. Pelletier M. R. Wentlandt K. Adamchik Y. Naus C. C. (2003). Epileptiform activity in hippocampal slice cultures exposed chronically to bicuculline: increased gap junctional function and expression. *J. Neurochem.* 86 687–699. 12859682 10.1046/j.1471-4159.2003.01893.x

[B54] Seifert G. Carmignoto G. Steinhäuser C. (2009). Astrocyte dysfunction in epilepsy. *Brain Res. Rev.* 63 212–221. 10.1016/j.brainresrev.2009.10.004 19883685

[B55] Seifert G. Carmignoto G. Steinhäuser C. Seifert G. Carmignoto G. Steinhäuser C. (2012). Astrocyte dysfunction in epilepsy. *Jasper’s Basic Mech. Epilepsies* 63 261–281.

[B56] Sosinsky G. E. Solan J. L. Gaietta G. M. Ngan L. Lee G. J. Mackey M. R. (2007). The C-terminus of connexin43 adopts different conformations in the Golgi and gap junction as detected with structure-specific antibodies. *Biochem. J.* 408 375–385. 10.1042/BJ20070550 17714073 PMC2267357

[B57] Steinhäuser C. Grunnet M. Carmignoto G. (2016). Crucial role of astrocytes in temporal lobe epilepsy. *Neuroscience* 323 157–169. 10.1016/j.neuroscience.2014.12.047 25592426

[B58] Steinlein O. K. (2004). Genetic mechanisms that underlie epilepsy. *Nat. Rev. Neurosci.* 5 400–408. 10.1038/nrn1388 15100722

[B59] Stout C. Charles A. (2003). Modulation of intercellular calcium signaling in astrocytes by extracellular calcium and magnesium. *Glia* 43 265–273. 10.1002/glia.10257 12898705

[B60] Szabó Z. Héja L. Szalay G. Kékesi O. Füredi A. Szebényi K. (2017). Extensive astrocyte synchronization advances neuronal coupling in slow wave activity in vivo. *Sci. Rep.* 7:6018. 10.1038/s41598-017-06073-7 28729692 PMC5519671

[B61] Tabernero A. Medina J. M. Giaume C. (2006). Glucose metabolism and proliferation in glia: role of astrocytic gap junctions. *J. Neurochem.* 99 1049–1061. 10.1111/j.1471-4159.2006.04088.x 16899068

[B62] Tovar K. R. Maher B. J. Westbrook G. L. (2009). Direct actions of carbenoxolone on synaptic transmission and neuronal membrane properties. *J. Neurophysiol.* 102 974–978. 10.1152/jn.00060.2009 19535488 PMC2724329

[B63] van de Bovenkamp-Janssen M. C. van der Kloet J. C. van Luijtelaar G. Roubos E. W. (2006). NMDA-NR1 and AMPA-GluR4 receptor subunit immunoreactivities in the absence epileptic WAG/Rij rat. *Epilepsy Res.* 69 119–128. 10.1016/j.eplepsyres.2006.01.003 16487682

[B64] Verkhratsky A. Rodríguez J. J. Parpura V. (2013). Astroglia in neurological diseases. *Future Neurol.* 8 149–158. 10.2217/fnl.12.90 23658503 PMC3645493

[B65] Verkhratsky A. Steinhäuser C. (2000). Ion channels in glial cells. *Brain Res. Rev.* 32 380–412. 10.1016/S0165-0173(99)00093-4 10760549

[B66] Vessey J. P. Lalonde M. R. Mizan H. A. Welch N. C. Kelly M. E. M. Barnes S. (2004). Carbenoxolone inhibition of voltage-gated ca channels and synaptic transmission in the retina. *J. Neurophysiol.* 92 1252–1256. 10.1152/jn.00148.2004 15028741

[B67] Wallraff A. Köhling R. Heinemann U. Theis M. Willecke K. Steinhäuser C. (2006). The impact of astrocytic gap junctional coupling on potassium buffering in the hippocampus. *J. Neurosci.* 26 5438–5447. 10.1523/JNEUROSCI.0037-06.2006 16707796 PMC6675300

[B68] Watanabe T. Morimoto K. Hirao T. Suwaki H. Watase K. Tanaka K. (1999). Amygdala-kindled and pentylenetetrazole-induced seizures in glutamate transporter GLAST-deficient mice. *Brain Res.* 845 92–96. 10529447 10.1016/s0006-8993(99)01945-9

[B69] Weber Y. G. Lerche H. (2008). Genetic mechanisms in idiopathic epilepsies. *Dev. Med. Child Neurol.* 50 648–654. 10.1111/j.1469-8749.2008.03058.x 18754913

[B70] Willoughby D. Thomas R. C. Schwiening C. J. (2001). The effects of intracellular pH changes on resting cytosolic calcium in voltage-clamped snail neurones. *J. Physiol.* 530 405–416. 10.1111/j.1469-7793.2001.0405k.x 11158272 PMC2278427

[B71] Xing L. Yang T. Cui S. Chen G. (2019). Connexin hemichannels in astrocytes: role in cns disorders. *Front. Mol. Neurosci.* 12:23. 10.3389/fnmol.2019.00023 30787868 PMC6372977

